# Predictive value of seizure onset for gross motor dysfunction in individuals with pathogenic *GABRB2* and *GABRB3* variants

**DOI:** 10.1002/epi.70096

**Published:** 2026-01-28

**Authors:** Sebastian Ortiz, Leonardo Affronte, Chiara Bagliani, Serene El‐Kamand, Anthony Sze Hon Kan, Isabel T. Kristoffersen, Rebekka S. Dahl, Anne F. Højte, Stéphane Auvin, Arjan Bouman, Shimriet Zeidler, Gerhard Kluger, Gaetan Lesca, Nicolas Chatron, Zeynep Goke‐Samar, Maria T. Papadopoulou, Matthildi Athina Papathanasiou Terzi, Elise Schaefer, Anne de Saint Martin, Sarah Baer, Mohammed Al Owain, Saud Takroni, Hesham Al‐Dhalaan, Paolo Bonanni, Alessandra Rossi, Nicoletta Zanotta, Marina Trivisano, Nicola Specchio, Angela de Dominicis, Pasquale Striano, Alessandro Orsini, Maria Margherita Mancardi, Sebastian Neuens, Melanie Jennesson‐Lyver, Ira Benkel‐Herrenbrueck, David Genevieve, Richard Sidlow, Kamer Tezcan, Ilona Krey, Johannes R. Lemke, Konrad Platzer, Damien Lederer, Inga Talvik, Ulvi Vaher, Kees P. J. Braun, Anne‐Marie Guerrot, Rebecca More, Matthias De Wachter, Sarah Weckhuysen, Evelina Carapancea, Maria Roberta Cilio, Julia Jacobs, Katalin Sterbova, Simona Balestrini, Renzo Guerrini, Giulio Peroni, Inger‐Lise Mero, Walaa ElNaggar, Nour Elkhateeb, Ariane Schmetz, Denise L. Chan, Ghayda M. Mirzaa, Boris Chaumette, Adrien Legrand, Amy McTague, Tommy Stödberg, Rebekah V. Harris, Samuel F. Berkovic, Ingrid E. Scheffer, Mary Chebib, Elena Gardella, Philip K. Ahring, Nathan L. Absalom, Rikke S. Møller

**Affiliations:** ^1^ Department of Epilepsy Genetics and Personalized Medicine Danish Epilepsy Centre, Filadelfia, Member of the European Reference Network EpiCARE Dianalund Denmark; ^2^ Department of Regional Health Research, Faculty of Health Sciences University of Southern Denmark Odense Denmark; ^3^ Child Neuropsychiatry IRCCS, Azienda Ospedaliero‐Universitaria di Bologna Bologna Italy; ^4^ Department of Neurosciences, Rehabilitation, Ophthalmology, Genetics, Maternal and Child Health (DINOGMI) University of Genoa Genoa Italy; ^5^ Child Neuropsychiatry Unit IRCCS, Istituto Giannina Gaslini, Full Member of the European Reference Network EpiCARE Genoa Italy; ^6^ School of Medical Sciences, Faculty of Medicine and Health, Brain and Mind Centre The University of Sydney Sydney New South Wales Australia; ^7^ INSERM NeuroDiderot, DMU Innov‐RDB, Neurologie Pédiatrique, AP‐HP, Hôpital Robert‐Debré, Member of European Reference Network EpiCARE Université de Paris Paris France; ^8^ Department of Clinical Genetics Erasmus University Medical Center Rotterdam The Netherlands; ^9^ Center for Pediatric Neurology, Neurorehabilitation, and Epileptology Schoen‐Clinic Vogtareuth Germany; ^10^ Children's Hospital Salzburg Research Institute “Rehabilitation, Transition and Palliation” PMU Salzburg Salzburg Austria; ^11^ Genetics Department Hospices Civils de Lyon, Member of ERN EpiCARE Lyon France; ^12^ Neuromyogene Institute, Pathology and Genetics of Neuron and Muscle, CNRS UMR 5261 INSERM U1315 Université Claude Bernard Lyon 1 Lyon France; ^13^ Department of Pediatric Clinical Epileptology, Sleep Disorders and Functional Neurology Hôpital Femme Mère Enfant, Member of ERN EpiCARE, University Hospitals of Lyon (HCL) Lyon France; ^14^ CNRS, INSERM, Centre de Recherche en Neurosciences de Lyon CRNL U1028 UMR5292, EDUWELL Université Claude Bernard Lyon 1 Bron France; ^15^ Service de Génétique Médicale Institut de Génétique Médicale d'Alsace Strasbourg France; ^16^ Department of Neuropediatrics Hôpitaux Universitaires de Strasbourg Strasbourg France; ^17^ Institute for Genetics and Molecular and Cellular Biology (IGBMC), CNRS UMR7104, INSERM U1258 University of Strasbourg Illkirch France; ^18^ French Reference Center for Rare Epilepsies (CréER), ERN EpiCARE France; ^19^ Department of Medical Genomics, Centre for Genomic Medicine King Faisal Specialist Hospital and Research Centre Riyadh Saudi Arabia; ^20^ Department of Neuroscience, Center for Autism Research King Faisal Specialist Hospital and Research Center Riyadh Saudi Arabia; ^21^ Epilepsy and Clinical Neurophysiology Unit, Scientific Institute for Research, Hospitalization and Healthcare IRCCS, E. Medea, IRCCS Eugenio Medea Conegliano Italy; ^22^ Unit of Clinical Neurophysiology and Epilepsy Centre IRCCS E. Medea Lecco Italy; ^23^ Neurology, Epilepsy and Movement Disorders Unit Bambino Gesù Children's Hospital, IRCCS, Full Member of European Reference Network EpiCARE Rome Italy; ^24^ Pediatric Neurology and Muscular Diseases Unit, Full Member of the European Reference Network EpiCARE IRCCS Istituto Giannina Gaslini Genoa Italy; ^25^ Pediatric Neurology University Hospital of Pisa, Azienda Ospedaliero Universitaria Pisana Pisa Italy; ^26^ Unit of Child Neuropsychiatry, Full Member ERN EpiCARE IRCCS Istituto Giannina Gaslini Genoa Italy; ^27^ Department of Genetics Hôpital Universitaire Des Enfants Reine Fabiola, Hôpital Universitaire de Bruxelles, Université Libre de Bruxelles Brussels Belgium; ^28^ Department of Pediatric Neurology American Memorial Hospital, CHU Reims Reims France; ^29^ Sana‐Krankenhaus Düsseldorf‐Gerresheim Academic Teaching Hospital der Heinrich‐Heine‐University Düsseldorf Düsseldorf Germany; ^30^ Department of Clinical Genetics, Inserm U1183, Centre de Référence «Anomalies du Développement et Syndromes Malformatifs» University Hospital of Montpellier, Montpellier University Montpellier France; ^31^ Department of Medical Genetics and Metabolism Valley Children's Hospital Madera California USA; ^32^ Department of Genetics Kaiser Permanente Sacramento California USA; ^33^ Institute of Human Genetics University of Leipzig Medical Center Leipzig Germany; ^34^ Centre de Génétique Humaine Institut de Pathologie et de Génétique Gosselies Belgium; ^35^ Tallin Children's Hospital Tallin Estonia; ^36^ Children's Clinic Tartu University Hospital Tartu Estonia; ^37^ Department of Child Neurology and Neurosurgery University Medical Center, Utrecht Brain Center, University Medical Center Utrecht, Utrecht University Utrecht The Netherlands; ^38^ Department of Genetics and Reference Center for Developmental Disorders, Inserm U1245 and CHU Rouen Univ Rouen Normandie, Normandie Univ Rouen France; ^39^ Département de Pédiatrie Néonatale Réanimation Pédiatrique Centre Hospitalier Universitaire de Rouen Rouen France; ^40^ Department of Pediatric Neurology Antwerp University Hospital, University of Antwerp Edegem Belgium; ^41^ Department of Neurology Antwerp University Hospital Antwerp Belgium; ^42^ VIB Center for Molecular Neurology Antwerp Belgium; ^43^ Translational Neurosciences, Faculty of Medicine and Health Science University of Antwerp Antwerp Belgium; ^44^ Institute of Neuroscience Université Catholique de Louvain Brussels Belgium; ^45^ Division of Pediatric Neurology, Department of Pediatrics Saint‐Luc University Hospital, Université Catholique de Louvain Brussels Belgium; ^46^ Hotchkiss Brain Institute and Alberta Children's Hospital Research Institute University of Calgary Calgary Alberta Canada; ^47^ Department of Paediatric Neurology, Second Faculty of Medicine Charles University and Motol Epilepsy Center, University Hospital Motol Prague Czech Republic; ^48^ Department of Clinical and Experimental Epilepsy UCL Queen Square Institute of Neurology London UK; ^49^ Neuroscience Department Meyer Children's Hospital, European Reference Network ERN EpiCARE Florence Italy; ^50^ Chalfont Centre for Epilepsy London UK; ^51^ Child and Adolescent Psychiatry Unit, Neuroscience and Human Genetics Department Meyer Children's Hospital IRCCS Florence Italy; ^52^ Department of Medical Genetics Oslo University Hospital Oslo Norway; ^53^ Department of Pediatrics, Faculty of Medicine Cairo University Giza Egypt; ^54^ Department of Clinical Genetics Cambridge University Hospitals NHS Foundation Trust Cambridge UK; ^55^ Institute of Human Genetics, Medical Faculty and University Hospital Düsseldorf Heinrich Heine University Düsseldorf Germany; ^56^ Neurology Department Sydney Children's Hospital Sydney New South Wales Australia; ^57^ School of Women's and Children's Health UNSW Medicine, UNSW Sydney Sydney New South Wales Australia; ^58^ Norcliffe Foundation Center for Integrative Brain Research Seattle Children's Research Institute Seattle Washington USA; ^59^ Department of Pediatrics University of Washington School of Medicine Seattle Washington USA; ^60^ Department of Laboratory Medicine and Pathology University of Washington School of Medicine Seattle Washington USA; ^61^ INSERM U1266, Institute of Psychiatry and Neuroscience of Paris Université Paris Cité Paris France; ^62^ GHU PARIS Psychiatrie et Neurosciences Sainte‐Anne Hospital Paris France; ^63^ Developmental Neurosciences UCL Great Ormond Street Institute of Child Health London UK; ^64^ Department of Neurology Great Ormond Street Hospital London UK; ^65^ Division of Pediatric Neurology, Department of Women's and Children's Health Karolinska Institutet Stockholm Sweden; ^66^ Section of Pediatric Neurology Astrid Lindgren Children's Hospital, Karolinska University Hospital Stockholm Sweden; ^67^ Epilepsy Research Centre, Department of Medicine Austin Health, University of Melbourne Heidelberg Victoria Australia; ^68^ Murdoch Children's Research Institute Parkville Victoria Australia; ^69^ Department of Paediatrics Royal Children's Hospital, The University of Melbourne Parkville Victoria Australia; ^70^ Florey Institute of Neuroscience and Mental Health Heidelberg Victoria Australia; ^71^ School of Medical Sciences, Faculty of Medicine and Health Brain and Mind Centre, The University of Sydney Camperdown New South Wales Australia; ^72^ School of Science Western Sydney University Penrith New South Wales Australia; ^73^ Behaviour and Development MARCS Institute for Brain Westmead New South Wales Australia

**Keywords:** clinical biomarker, developmental and epileptic encephalopathy, functional variant classification, GABA_A_ receptor–related disorders, genotype–phenotype correlation, motor disability prediction

## Abstract

**Objective:**

Pathogenic variants in γ‐aminobutyric acid type A (GABA_A_) receptor genes have been associated with a wide spectrum of neurological disorders. We aimed to delineate the clinical trajectories associated with gain‐of‐function (GoF) and loss‐of‐function (LoF) variants in *GABRB2* and *GABRB3*, and to develop a risk‐prediction model for gross motor dysfunction based on age at seizure onset.

**Methods:**

Clinical data, including seizure onset, epilepsy syndromes, cognitive outcomes, and gross motor function classification system (GMFCS), were collected through direct interviews, physician reports, and literature review. Kruskal–Wallis, Mantel–Cox and non‐parametric analysis of variance (ANOVA) with Dunn's corrected post hoc tests were used for statistical comparisons. A logistic ordinal regression model was developed to predict GMFCS outcomes based on age at seizure onset.

**Results:**

We analyzed a cohort of 117 individuals with pathogenic *GABRB2* (*n* = 49) and *GABRB3* (*n* = 68) variants. Fifty‐three individuals carried GoF variants and 64 carried LoF variants. The GoF group was associated with earlier seizure onset, higher seizure frequency, and lower rates of seizure freedom. Gross motor dysfunction was markedly worse in the GoF group, with 64% classified as GMFCS IV or V (non‐ambulation), compared to 7.5% in the LoF group. An inverse correlation was found between age at seizure onset and GMFCS severity in the GoF, but not the LOF group. The risk model predicted a >90% likelihood of non‐ambulation for individuals with GoF variants and seizure onset before 1 month of age, decreasing to ~35% with seizure onset after 20 months.

**Significance:**

We found a clear genotype–phenotype correlation in *GABRB2‐* and *GABRB3*‐related disorders, demonstrating that GoF variants are associated with a more severe neurodevelopmental trajectory. The age at seizure onset serves as a biomarker for predicting motor outcomes in individuals with GoF variants. These findings provide guidance regarding prognosis, need for early intervention, and data for comparison of efficacy in targeted therapeutic interventions for GABA_A_ receptor–related disorders.

**Key points**

- Gain‐of‐function (GoF) variants in *GABRB2* and *GABRB3* lead to earlier seizure onset and more severe neurodevelopmental outcomes than loss‐of‐function variants.
- An earlier age at seizure onset correlates with more severe gross motor dysfunction in individuals with *GABRB2* or *GABRB3* GoF variants.
- Functional variant classification and seizure‐onset age guide prognosis and serve as comparators for future clinical trial design.

## 1 INTRODUCTION

Developmental and epileptic encephalopathies (DEEs) are the most severe group of epilepsies, with a high proportion having a monogenic etiology. More than 900 genes have been associated with genetic DEEs,^1^, ^2^ including genes encoding the γ‐aminobutyric acid type A (GABA_A_) receptor.^3^ GABA_A_ receptors are ligand‐gated ion channels that facilitate neuronal inhibition by opening a chloride‐permeable pore in response to GABA binding. Structurally, these receptors are pentameric assemblies, and their composition varies widely due to the availability of 19 different subunits (α1–6, β1–3, γ1–3, δ, ε, θ, π, and ρ1–3). In humans, the most frequent receptor consists of two α, two β, and one γ2 subunit.^4^

Pathogenic variants in the *GABRB2* and *GABRB3* genes, encoding the β2 and β3 subunits of the GABA_A_ receptor, respectively, have been associated with a spectrum of neurodevelopmental disorders and epilepsies.^5^, ^6^, ^7^, ^8^, ^9^ Loss‐of‐function (LoF) and gain‐of‐function (GoF) variants have been reported, and associated phenotypes are linked to the functional outcome of the variants. LoF variants are typically associated with seizure onset after 3 months, febrile seizures, milder cognitive, motor and epilepsy outcomes.^5^, ^8^, ^10^ In contrast, GoF variants have been associated with early infantile developmental and epileptic encephalopathies (EIDEEs) with onset before 3 months of age and severe neurological comorbidities, including motor and cognitive impairment, hyperkinetic movement disorders, feeding difficulties and higher risk of early death.^5^, ^6^, ^7^, ^9^, ^11^

Although previous studies have demonstrated that epilepsy and developmental outcomes vary widely between patients with GoF and LoF variants, the use of relatively broad phenotypic descriptions and limited information on longitudinal trajectories have left critical aspects unexplored. For example, understanding motor and cognitive outcomes and other morbidities, in addition to epilepsy prognosis, is critical to determine the efficacy of treatment and allow comprehensive clinical trial designs with meaningful therapeutic endpoints.

This study will systematically examine the relationship between age at seizure onset and gross motor outcome in individuals with *GABRB2* and *GABRB3* pathogenic variants. Unlike prior GABA_A_ receptor studies that focused primarily on epilepsy phenotypes and cognitive outcomes, we focus on motor dysfunction as a distinct, quantifiable clinical endpoint. We identify an association between age at seizure onset and severity of gross motor dysfunction and develop a risk‐prediction model to inform clinical diagnosis, prognosis of motor development, and treatment evaluation.

## 2 METHODS

### 2.1 Patient cohort and data collection

One hundred and seventeen individuals with pathogenic variants in *GABRB2* (*n* = 49) and *GABRB3* (*n* = 68) were included. Forty individuals were novel, and updated clinical information was provided for a substantial number of the previously published cases.^5^, ^6^, ^7^, ^8^, ^9^, ^11^, ^12^, ^13^, ^14^, ^15^, ^16^, ^17^, ^18^ New cases were collected via international patient advocacy groups (Cure GABA‐A and GABA‐Alliance), the European Reference Network for all rare and complex epilepsies (ERN‐EpiCARE) Genetic platform (https://epi‐care.eu/collaborative‐genetic‐research/), or via an international network of epilepsy and genetics centers in Europe, North America, and Australia.

Demographic, genetic, and clinical information was collected, including information on pregnancy, birth, neurodevelopmental milestones, neurodevelopmental/cognitive outcomes, age at seizure onset, seizure types, epilepsy syndromes, and seizure outcomes. Developmental and cognitive levels were based on clinical and, whenever available, standardized assessment. The Diagnostic and Statistical Manual of Mental Disorders, Fifth Edition (DMS‐5), was used to classify the findings. Developmental delay (DD) was classified as mild, moderate, or severe in children who were 5 years of age or younger. Individuals older than 5 years were classified as having mild, moderate, severe, or profound intellectual disability (ID). During video interview, we obtained information about gross motor function and movement disorders. All data were collected and stored at the Danish Epilepsy Centre. Semiology of the first seizure and epilepsy syndrome were classified according to the International League Against Epilepsy (ILAE) classification,^19^, ^20^, ^21^, ^22^, ^23^ whenever possible.

### 2.2 Gross motor function classification system (GMFCS)

Gross motor function classification system (GMFCS)^24^ scores were determined through interviews with families and/or physicians for all new and some previously published individuals. For published individuals, where more recent clinical details were not available, information was extracted from the articles.

Individuals were categorized into three GMFCS groups: (1) those who walk independently (GMFCS I and II); (2) those who walk with support (GMFCS III); and (3) those who cannot walk independently, regardless of head control (GMFCS IV and V). Individuals with normal gross motor function were not classified with GMFCS.

### 2.3 Inclusion criteria

**FIGURE 1:**
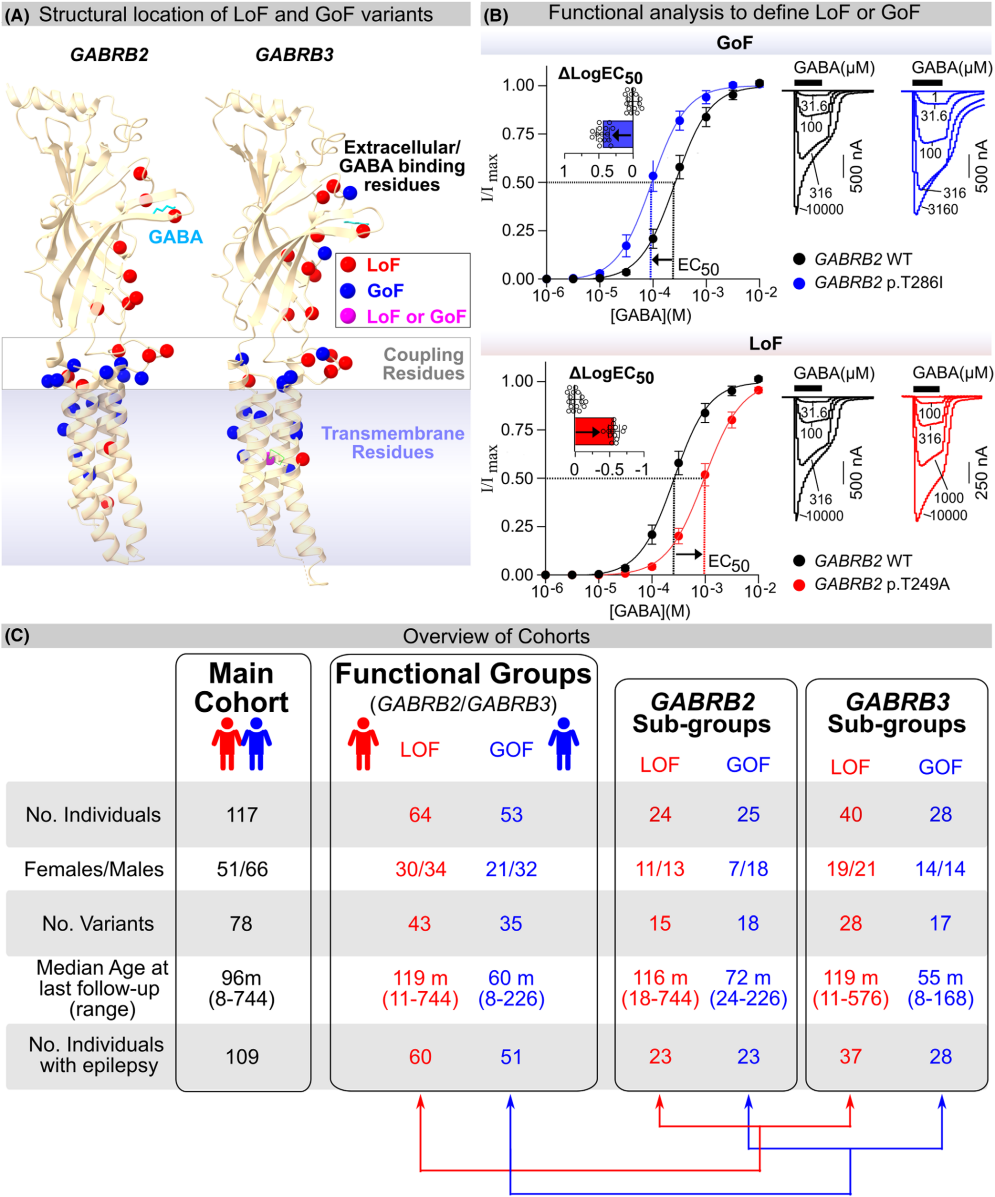
Overview of cohort segregation into both functional loss of function (LoF) and gain of function (GoF) groups and gene sub‐groups compared in this study. (A) Location of *GABRB2* and *GABRB3* variants in the structure of the β2 (pdb:8dd3) and β3 (pdb:6hup). The γ‐aminobutyric acid (GABA) molecule bound is shown in aqua, location of LoF variants depicted as red spheres, GoF variants as blue, and residues where different amino acid changes result in either LoF or GoF in purple. Variants in both genes are roughly similarly spread between the extracellular and GABA‐binding regions, coupling residues, and transmembrane domain. (B) Functional analysis of variants depicting a GoF *GABRB2* p.Thr286Ile [blue] and a LoF *GABRB2* p.Thr294Ala [red] variant). Concentration–response curves (left) were constructed to determine the ΔlogEC_50_ change between variant and wild‐type receptors recorded on the same day with a minimum *n* = 10. The ΔlogEC_50_s were compared with a one‐way analysis of variance (ANOVA) and Dunnett's post hoc test (inset). Variants were assigned as GoF if GABA sensitivity significantly increased and LoF if it decreased. Representative traces (*right*) are shown of responses to GABA activation of concatenated receptors containing a single variant copy. (C) Summary of the general features of the main cohort, the two LoF and GoF variant patient groups based on functional change (above), and the four gene‐specific LoF and GoF sub‐groups with *GABRB2* or *GABRB3* gene variants (*right*).

Only individuals with complete clinical data, as well as information on the functional effect of the variant, were included. The functional effect of 54 variants was reported previously^5^, ^8^; the same methods were used to functionally assess 17 additional variants for this study (Figure 1). The variants were grouped into GoF or LoF based on GABA sensitivity.

### 2.4 Functional characterization

76.4% of the individuals harboured variants that were either null variants or missense variants that have previously been functionally characterized.^5^, ^8^ For the remaining 17 missense variants identified in 22 individuals, functional evaluation of variant receptors was performed using a custom made two‐electrode voltage clamp apparatus described previously.^5^, ^8^, ^25^ Briefly, concatenated pentameric receptor constructs using human GABA_A_ receptor subunits were used that allowed for the systematic introduction of a point‐mutated subunit into the second position of a γ2‐β2‐α1‐β2‐α1 or γ2‐β3‐α1‐β3‐α1 construct. Mutant constructs were made and verified by sequencing followed by sub‐cloning into the concatenated construct using standard restriction digestion and ligation. Linearized complementary DNA (cDNA) was generated and cRNA for each concatenated receptor construct was produced using the mMessage mMachine T7 Transcription Kit (Thermo Fisher).

The complementary RNA (cRNAs) of wild‐type (WT) and mutant concatenated receptors were injected into *Xenopus laevis* oocytes at ~25 ng cRNA per oocyte. Oocytes were incubated for 2 days at 18°C in modified Barth's solution (96 mM NaCl, 2 mM KCI, 1 mM MgCl_2_, 1.8 mM CaCl_2_, 5 mM HEPES, 2.5 mM sodium pyruvate, 0.5 mM theophylline, and 100 mg/L gentamicin; pH 7.4). All recordings were performed at room temperature. Oocytes were placed in a recording chamber, and ringer (ND96) solution (96 mM NaCl, 2 mM KCl, 1 mM MgCl_2_, 5 mM HEPES, 1.8 mM CaCl_2_, pH 7.4) was continuously perfused. The pipettes were backfilled with 3 M KCl and had open pipette resistances from 0.4 to 2 MΩ when submerged in ND96 solution. Oocytes were voltage clamped using an Axon GeneClamp 500B amplifier (Molecular Devices) at a holding potential of −60 mV. Amplified currents were low‐pass filtered at 20 Hz using a four‐pole Bessel filter (Axon GeneClamp 500B), digitized using a Digidata 1440 (Molecular Devices), and sampled at 200 Hz on a personal computer using the pClamp 10.2 suite (Molecular Devices). Episodic traces following triggering events representing responses to individual applications were collected.

On each experimental day, the functional properties of WT receptors were assessed along with the mutant receptors to eliminate the impact of inter‐day variation and variation between batches of oocytes. To assess maximum current amplitudes, 10 mM GABA was applied. GABA concentration–response relationships were then determined by applications of increasing concentrations of GABA to the oocyte. Final datasets for GABA concentration–response were collected from at least 10 independent experiments performed on at least two different batches of oocytes.

Raw traces were analyzed using pClamp 10.2. To determine the half‐maximal effective concentration (EC_50_) values of GABA concentration–response relationships, the Hill equation was fitted to peak GABA‐evoked current amplitudes for individual oocytes using GraphPad Prism 10:

$$I=\mathrm{Abs}.I_{\max}\left({\left[A\right]}^{\mathrm{nH}}/\left({\left[A\right]}^{\mathrm{nH}}+{\left[{\mathrm{EC}}_{50}\right]}^{\mathrm{nH}}\right)\right)$$

where $\mathrm{Abs}.I_{\max}$ is the absolute maximum current, ${\mathrm{EC}}_{50}$ is the concentration that evokes half‐maximum response, $\left[A\right]$ is the ligand (GABA) concentration, and nH is the Hill slope. For each individual oocyte, a complete concentration–response curve was recorded as a single determination (*n*). From the ${\mathrm{EC}}_{50}$ value the corresponding $\log{\mathrm{EC}}_{50}$ value was calculated. By fitting the Hill equation to all data for each construct, final ${\mathrm{EC}}_{50}$ values were calculated. For each experimental day, the mean $\log{\mathrm{EC}}_{50}$ for WT construct ($\log{\mathrm{EC}}_{50,\mathrm{wt}}$) was calculated. In addition, the $∆\log{\mathrm{EC}}_{50}$ value for each oocyte containing a mutant construct tested on the same day was calculated using the following equation:

$$∆\log{\mathrm{EC}}_{50}=\log{\mathrm{EC}}_{50,\mathrm{wt}}-\log{\mathrm{EC}}_{50}$$

Variants were defined as GoF if the ΔlogEC_50_ was >0.2 and *p* < 0.0001, and LoF where the ΔlogEC_50_ was <‐0.2 and *p* < 0.0001, when compared by one‐way analysis of variance (ANOVA) with Dunnett's post hoc test.

The normalized maximum GABA‐evoked current amplitude ($I_{\max}$) was calculated using the peak current evoked by 10 mM GABA at WT controls ($\mathrm{Abs}.I_{\max,\mathrm{wt}}$) and mutants $\left(\mathrm{Abs}.I_{\max}\right)$ for parallel experiments performed on the same experimental day. To determine the $(I_{\max}$) for each individual experiment on a variant following equation was used:

$$I_{\max}=\frac{\mathrm{Abs}.I_{\max}}{\mathrm{Abs}.I_{\max,\mathrm{wt}}}$$

Variants were defined as LoF based on the *I*_max_ where the reduction was >50% and *p* < 0.0001 when compared with a Mann–Whitney test to WT.

### 2.5 Data analysis and statistics

The age at seizure onset, GMFCS, and cognitive scores were compared with a Kruskal–Wallis non‐parametric ANOVA and Dunn's corrected post hoc test. Age at seizure onset was compared with a Mantel–Cox test.

A simple risk model to predict gross motor function was developed with a logistic ordinal regression of the log(age at onset) at different GMFCS scores in SPSS. The resultant outputs were converted into cumulative risk percentages for GMFCS scores at different ages at onset.

### 2.6 Ethics statement

The study was conducted according to the ethical principles for medical research outlined in the Declaration of Helsinki. The study was approved by the Institutional Review Board at the Danish Epilepsy Centre, under protocol number EMN‐20240‐1998. All individuals or legal guardians provided written informed consent for research participation, and the appropriate institutional forms have been archived.

## 3 RESULTS

### 3.1 Demographics

We collected a cohort of 117 individuals (51 female, 66 male) with neurodevelopmental disorders due to pathogenic variants in *GABRB2* (49 individuals) and *GABRB3* (68 individuals). In the 49 individuals with a *GABRB2* variant, 33 heterozygous missense variants were identified, whereas 37 missense and 8 null variants (4 frameshift, 1 exon 1–3 deletion, 1 whole gene deletion, and 2 splice‐site variants) were identified in the 68 individuals with a *GABRB3* variant. According to American College of Medical Genetics (ACMG) criteria, variants were classified as pathogenic (44), likely pathogenic (30), or variants of unknown significance^5^ (Table S1).

### 3.2 Functional characterization of novel variants

Fifty‐five variants had been characterized previously (24 in *GABRB2* and 31 in *GABRB3*).^5^, ^8^ Ten *GABRB2* and seven *GABRB3* variants were functionally assessed in this study using two‐electrode voltage clamp. Twelve of the novel variants were characterized as LoF and five as GoF (Tables S1 and S2). Eight null variants were not tested, as they were assumed to be LoF. All mutant receptors showed concentration‐dependent increases in response to GABA applications. Maximal GABA‐evoked current amplitudes were evaluated for receptors containing the 17 subunit variants (10 β2 and 7 β3). After functional testing was performed, all variants were then reclassified as pathogenic or likely pathogenic according to the ACMG criteria^26^ (Table S1). Examples of functional testing are provided in Figure 1 (GoF β2 T286I and LoF β2 T249A). Expanded functional and phenotypical information is provided in Figure 1 and Tables S1 and S2.

### 3.3 Follow‐up and mortality

The median age at last follow‐up was 96 months (interquartile range [IQR]: 48–148; range: 8 months to 62 years). Only one patient had perinatal complications following a premature birth (29 weeks) that may have contributed to her phenotype. Eight individuals (seven GoF and one LoF) had died (two *GABRB2* and six *GABRB3*), with the age at death available for six; the average age at death was 47 months (range: 24–72 months). Three individuals (including one individual with an LoF variant) died from possible sudden unexpected death in epilepsy (SUDEP), one died due to respiratory failure following a respiratory infection, and one died due to super refractory status epilepticus, whereas the cause of death was unknown in the other three cases.

### 3.4 Cohort segregation: LoF And GoF functional groups and *GABRB2*/*GABRB3* sub‐groups

We first segregated the cohort into two groups, based on the functional change of the variants (LoF or GoF) (Figure 1). Sixty‐four individuals had 43 different LoF variants. The median age at last follow‐up in the LoF group was 119 months (range: 11–744 months), and 94% (60/64) of the individuals had epilepsy. Fifty‐three individuals had 35 different GoF variants. The median age at last follow‐up in the GoF group was 60 months (range_ 8–226 months), and 96% (51/53) had epilepsy. We then divided these groups according to gene and function, making four subgroups: *GABRB2* LoF and GoF and *GABRB3* LoF and GoF (Figure 1). The subgroups did not differ substantially in number of patients, sex, or age at last follow‐up.

### 3.5 Epilepsy and seizure outcomes of patients with LoF and GoF variants

**FIGURE 2:**
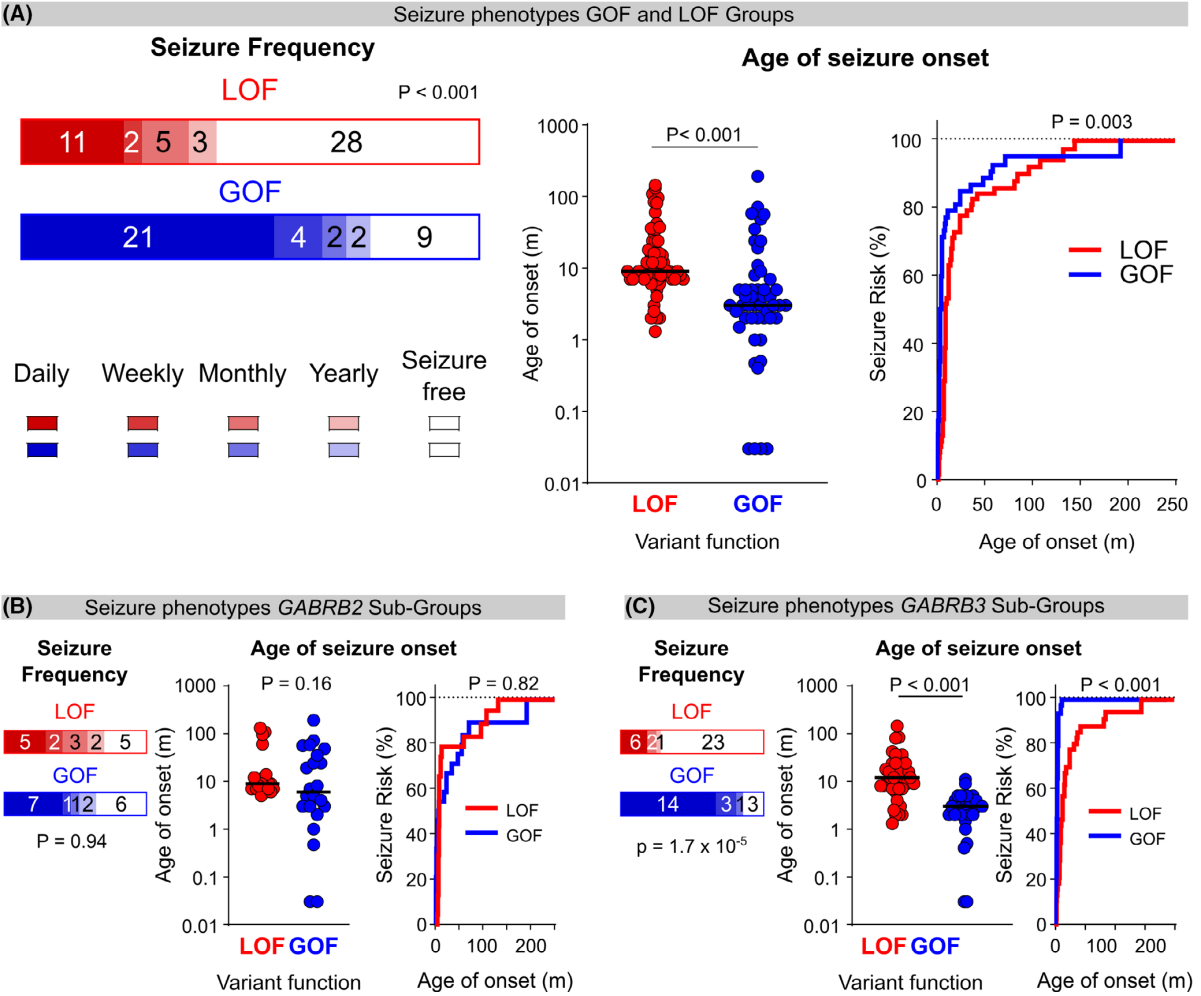
Seizure frequency and age at onset at LoF and GoF variant groups. (A) Selected clinical parameters of the seizure phenotype were assessed for their association with their molecular phenotype at LoF and GoF variants. Bar graph (*left*) depicting seizure frequency at LoF (red) and GoF (blue), with shading from darkest to lightest to denote daily, weekly, monthly, or yearly seizures, respectively. Seizure‐free individuals are depicted in white (*p* < 0.001, Mann–Whitney test, *χ* ^2^ = 548.5; *n* = 49 LoF and 38 GoF). Age at seizure onset (*middle*) is shown on a log scale with LoF depicted as red dots, GoF as blue, and the median is a solid bar (*p* < 0.001, Mann–Whitney test, *χ* ^2^ = 698; *n* = 59 LoF and 50 GoF). Seizure risk (*right*) is shown for LoF (red line) and GoF (blue line) and *p*‐value for a Mantel–Cox test is shown. “m” refers to months on the *y*‐axis. (*p* = 0.003, Mantel–Cox test; *n* = 62 LoF and 52 GoF). (B) No association of seizure frequency or age oat seizure onset between gene‐specific *GABRB2* LoF and GoF subgroups (seizure frequency: *p* = 0.94, *χ* ^2^ = 141, *n* = 17 LoF and 17 GoF; age at onset *p* = 0.16, *χ* ^2^ = 181.5, *n* = 22 LoF and 22 GoF, Mann–Whitney test, *p* = 0.82, Mantel–Cox test, *n* = 23 LoF and 24 GoF). (C) Association of GoF with increased seizure frequency and younger age at seizure onset between gene‐specific *GABRB3* LoF and GoF subgroups (seizure frequency: *p* < 0.001, *χ* ^2^ = 128.5, *n* = 32 LoF and 21 GoF; age at onset *p* < 0.001, *χ* ^2^ = 137.5, *n* = 37 LoF and 28 GoF, Mann–Whitney test, *p* < 0.001, Mantel–Cox test, *n* = 39 LoF and 28 GoF).

The GoF group had a lower median age at seizure onset (3 months, 95% confidence interval [CI]: 2.5–5) compared with the LoF group (9 months, CI: 8–12; *p* < 0.001). In addition, individuals with a GoF variant had more frequent seizures (*p* < 0.001), with daily seizures being more common in the GoF (42%) compared to the LoF group (19%). Similarly, seizure freedom was less frequently achieved in the GoF (15%) than the LoF group (45%) (Figure 2A).

Epilepsy syndromes in the LoF group included 48.2% (28/58) with a DEE, including 1 case with infantile epileptic spasms syndrome (IESS), 1 with early infantile developmental and epileptic encephalopathy (EIDEE), 13% (8/58) with a phenotype within the Dravet syndrome–like (3 individuals) and genetic epilepsy with febrile seizures plus (GEFS+) spectrum (5 individuals), 13% (8/58) with epilepsy with myoclonic–atonic seizures (EMAtS), 7% (4/58) with focal epilepsy, and 3.5% (2/58) with idiopathic generalized epilepsy (one with childhood absence epilepsy [CAE] and one with juvenile myoclonic epilepsy [JME]). The epilepsy syndrome was not classifiable in the remaining 13% (8/58).

Epilepsy syndromes in the GoF group included EIDEE in 23.5% (12/51), epilepsy of infancy with migrating focal seizures (EIMFS) in 17.6% (9/51), and 4% (2/51 cases) with each of the following syndromes: DEE with spike–wave activation during sleep (DEE‐SWAS), IESS and Lennox–Gastaut syndrome (LGS), and the remaining 47% (24/51) had unclassified DEE.

For *GABRB2*, age at seizure onset was similar for the LoF (9 months, CI: 7–12) and GoF (6 months, CI: 3–35) subgroups (*p* = 0.94 Mann–Whitney test, *p* = 0.82 Mantel–Cox test), as was seizure frequency (*p* = 0.95). Seizure freedom occurred in 26% (11/43) of individuals, of whom 5 had LoF and 6 had GoF variants (Figure 2B). However, patients with *GABRB2* GoF variants had a broader range of age at seizure onset than those with LoF variants, with a much greater variance for the cohort (*p* < 0.001, *F*‐test log_10_(age at onset)). By contrast, individuals with *GABRB3* GoF variants had a higher seizure frequency (*p* < 0.001) and lower median age at seizure onset at 3 months (CI: 2–4) than those with LoF variants at 12 months (CI: 8–16), (*p* < 0.001 Mann–Whitney test, *p* < 0.001 Mantel–Cox test). Seizure freedom occurred in 62% (23/37) of individuals with *GABRB3* LoF and 10.7% (3/28) of those with *GABRB3* GoF variants (Figure 2C).

Taken together, phenotypes of individuals with GoF variants were more severe than those with LoF variants. However, when studying each gene individually, there is a wider range of clinical variability for individuals with *GABRB2* GoF variants compared to *GABRB3* GoF variants.

### 3.6 Cognitive outcome is markedly worse for individuals with GoF variants

**FIGURE 3:**
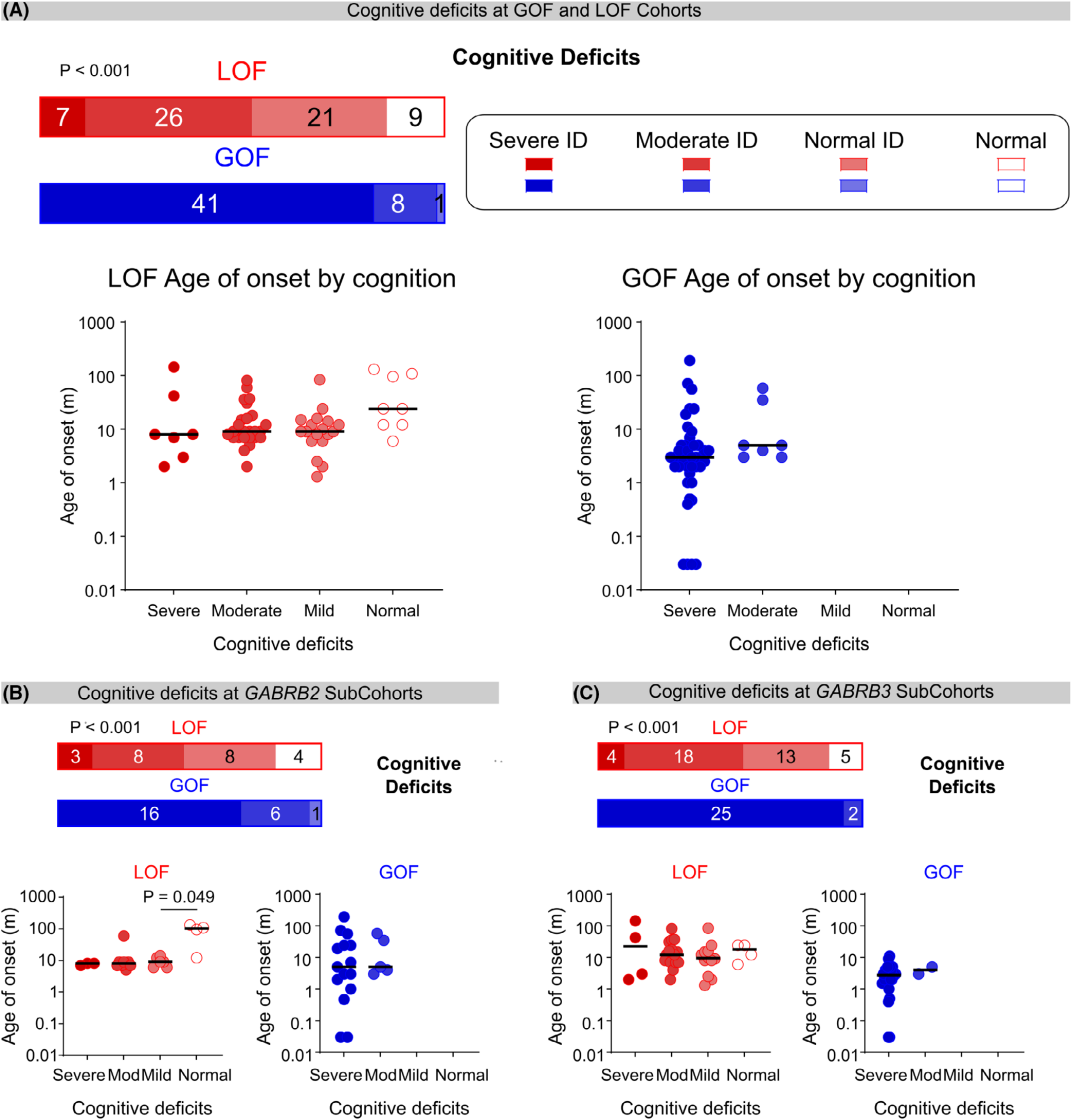
Cognitive impairment at LoF and GoF variants. (A) Comparison of cognitive deficits at LOF (red) and GOF (blue) variants. Bar graph (*top*) depicting cognitive impairment at LoF (red) and GoF (blue), with shading from darkest to lightest to denote severe, moderate, or mild intellectual disability, respectively. Individuals without intellectual disability are depicted in white (*p* < 0.001, *χ* ^2^ = 350.5, *n* = 63 LoF and 51 GoF, Mann–Whitney test). Age at seizure onset for LoF (*below left*) and GoF (*below right*) variants are shown; each individual is represented as a single dot with the same color scheme as above. Bars indicate median scores (LoF *p* = 0.11, H_4,58_ = 5.967, *n* = 58, Kruskal–Wallis test) or GoF (*p* = 0.072, *χ* ^2^ = 82, *n* = 48, Mann–Whitney test). (B) Comparison of cognitive deficits at *GABRB2* LoF and GoF sub‐groups for cognitive deficits (*GABRB2*: *p* < 0.001, *χ* ^2^ = 81, *n* = 23 LoF and 23 GoF, Mann–Whitney test) and age at seizure onset for different severity of cognitive deficits at *GABRB2* LoF (*p* = 0.049, Dunn's post hoc test, *p* = 0.036 H_4,21_ = 8.553, *n* = 21, Kruskal–Wallis test) and *GABRB2* GoF sub‐groups (*p* = 0.51, *χ* ^2^ = 29.5, *n* = 20, Mann–Whitney test). (C) Comparison of cognitive deficits at *GABRB3* LoF and GoF sub‐groups for cognitive deficits (*p* < 0.001, *χ* ^2^ = 78, *n* = 40 LoF and 28 GoF, Mann–Whitney test) and age at seizure onset for different severity of cognitive deficits at *GABRB3* LoF (*p* = 0.86, H_4,37_ = 0.07493, *n* = 37 Kruskal–Wallis test) and *GABRB3* GoF sub‐groups (n.d, too few data in moderate ID).

The level of cognitive or neurodevelopmental impairment in patients <5 years of age, was assessed using the DSM‐5, Text Revision^27^ or clinical observations where cognitive measurements were not available. Individuals in the GoF group were more severely impaired, as severe intellectual disability was observed more frequently (82%), compared with those in the LoF group (11%) (*p* < 0.001) (Figure 3A). Individuals with seizure onset before 1 month of age were found only in the GoF group and uniformly had severe‐profound intellectual disability. No association between the age at seizure onset and the severity of cognitive impairment (*p* = 0.11 and 0.072) was observed in either the LoF or GoF groups. For individuals with GoF variants, this was partly due to the very high proportion of GoF individuals with severe or profound intellectual disability (Figure 3A).

For both *GABRB2* or *GABRB3*, individuals with GoF variants had worse cognitive deficits (70% *GABRB2*, 93% *GABRB3*) than those with LoF variants (13% *GABRB2*, 10% *GABRB3*), (*GABRB2*: *p* < 0.01; *GABRB3*: *p* < 0.01), and were more likely to have severe intellectual disability (Figure 3B,C). There was no association between the age at seizure onset and severity of intellectual disability in patients with *GABRB2* GoF (*p* = 0.51) or *GABRB3* LoF (*p* = 0.86) variants.

Taken together, severe to profound intellectual disability was more frequent in individuals with GoF variants, and ubiquitous in GoF individuals with seizure onset in the first month of life.

### 3.7 Gross motor function is associated with age at seizure onset for individuals with GoF variants

Gross motor function was evaluated using GMFCS to determine each patient's abilities in terms of head control, sitting, and walking, and which equipment or mobility aids each patient might require. Scores were grouped into four categories: normal, GMFCS I and II, GMFCS III, and GMFCS IV and V.

**FIGURE 4:**
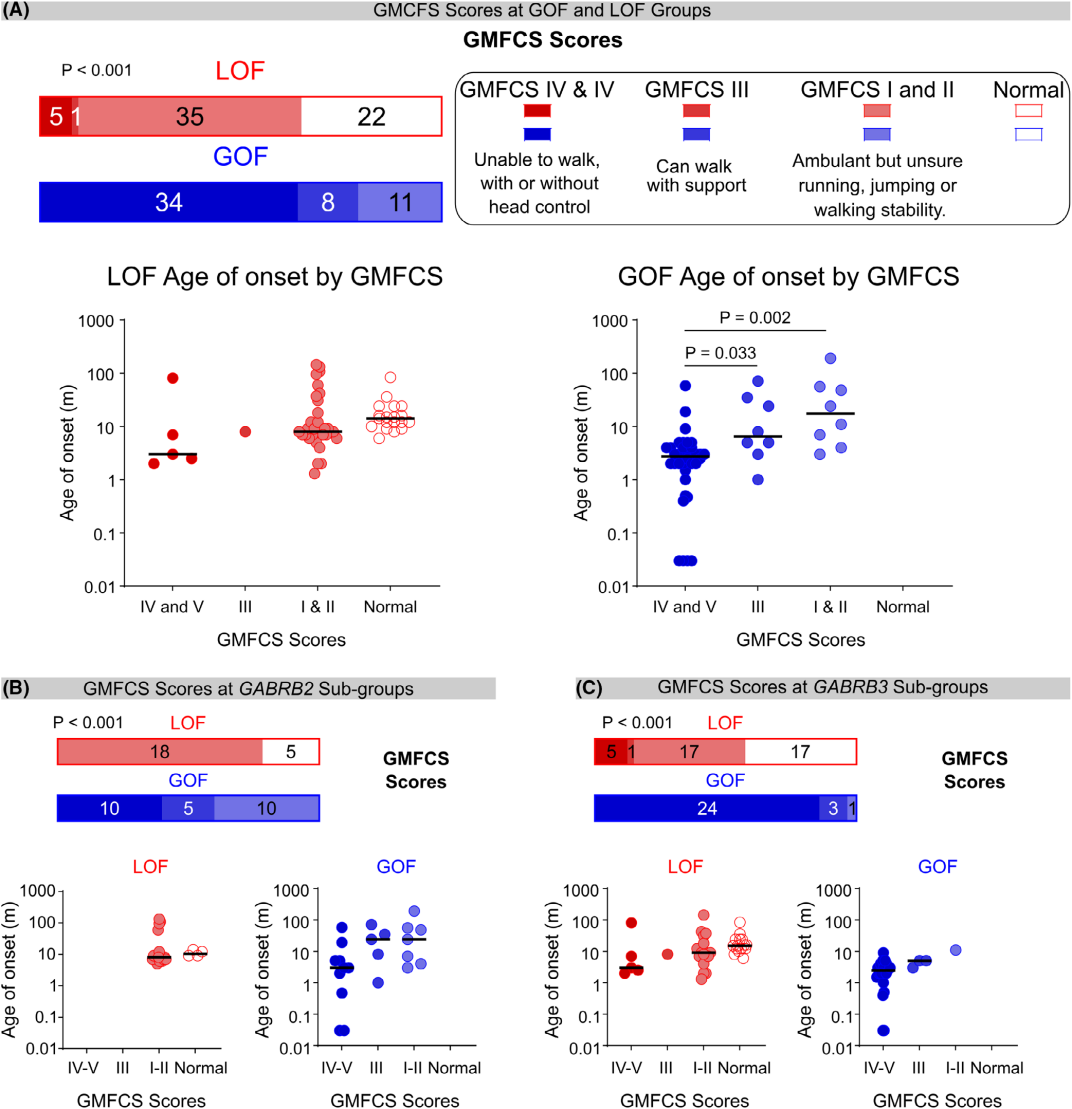
Gross motor function classification system (GMFCS) scores at LoF and GoF variants. (A) Bar graph (*top*) depicting gross motor deficits at LoF (red) and GoF (blue), with shading from darkest lightest to denote GMFCS IV and V (unable to walk with or without head control), GMFCS III (can walk with support), or GMFCS I and II (can walk without support but unsure running, jumping, or walking stability), respectively. Individuals with no gross motor dysfunction are depicted in white (*p* < 0.001, *χ* ^2^ = 387.5, *n* = 63 LoF and 53 GoF, Mann–Whitney test). Age at onset is not correlated with the GMFCS score for the LoF group (*below left*) (*p* > 0.05, *n* = 58, Dunn's post hoc tests) but is correlated with the group GOF (*below right*) (*p* < 0.001, H_3,50_ = 15.5, Kruskal–Wallis test; *p* = 0.033, *n* = 34 (IV and V) 8 (III), and *p* = 0.002, *n* = 34 (IV and V), 8 (III), Dunn's post hoc test), variants are shown with the same color scheme as above. (B) Gross motor deficits are less severe overall at the *GABRB2* LoF sub‐group than the GoF subgroup (*p* < 0.001, *χ* ^2^ = 90, *n* = 23 LoF and 25 GoF, Mann–Whitney test). No association was found between GMFCS classification and the age at onset in the *GABRB2* LoF (*p* = 0.24, *χ* ^2^ = 20.5, *n* = 21, Mann–Whitney test) or GoF subgroups (*p* = 0.063, H_3,22_ = 5.368, Kruskal–Wallis test). (C) Gross motor deficits are less severe overall at the *GABRB3* LoF sub‐group than the GoF subgroup (*χ* ^2^ = 91, *n* = 40 LoF and 28 GoF, Mann–Whitney test). No association was found between GMFCS classification and the age at onset in the *GABRB3* LoF (*p* = 0.14, H_4,37_ = 5.585, Kruskal–Wallis test) or GoF subgroups (*p* = 0.054, H_3,28_ = 5.824, Kruskal–Wallis test).

Overall, the GoF group had worse GMFCS scores than the LoF group (*p* < 0.001), (Figure 4A). Individuals with GoF variants were more likely to be non‐ambulant (GMFCS IV or V) (64%) than those with LoF (7.5%) variants. Moreover, younger age at seizure onset was associated with more severe levels of gross motor dysfunction in individuals with GoF variants (*p* < 0.001), with GMFCS IV and V having a younger age at seizure onset (median 2.8 months, 95% CI: 2–4) than those with GMFCS III (8 months, CI: 1–71) and GMFCS I and II (17.5 months, CI: 3–92) (*p* = 0.033 and *p* = 0.002, respectively) (Figure 4A). We found no difference between age at seizure onset and GMFCS scores in patients with LoF variants (*p* > 0.05).

Regardless of whether the variant was in the *GABRB2* or *GABRB3* gene, individuals with a GoF variant were more likely to be non‐ambulatory with a GMFCS IV or V score (40% *GABRB2*, 86% *GABRB3*) compared to those with a LoF variant (0% *GABRB2*, 13% *GABRB3*). Reflecting that individuals with GoF variants had worse GMFCS scores overall (*GABRB2* and *GABRB3*: *p* < 0.001) (Figure 4B,C).

No association was found between GMFCS classification and age at seizure onset, regardless of whether patients had LoF (*GABRB2 p* = 0.24, *GABRB3* 0.14) or GoF (*GABRB2 p* = 0.063, *GABRB3* 0.054) variants (Figure 4B,C). The median age at seizure onset for individuals with GMFCS IV and V was similar for the *GABRB2* GoF subgroup (3 months, CI: 8.5–36) and *GABRB3* GoF subgroup (2.5, CI: 1.6–4). We therefore developed a risk‐prediction model for gross motor dysfunction based on the age at seizure onset.

### 3.8 A simple risk‐prediction model for gross motor dysfunction severity based on age at seizure onset for patients with GoF variants

**FIGURE 5:**
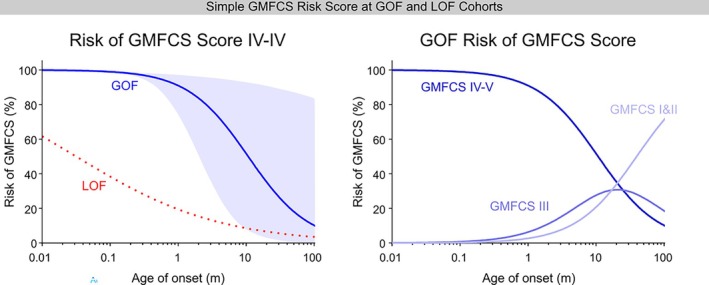
Simple model for gross motor function risk. Ordinal regression of GMFCS risk scores against the log age at onset. Best‐fit risk of GMFCS IV and V (*left*) at different ages at seizure onset shown for LoF (dotted red line) and GoF (blue line). GMFCS IV and V risk curve for GOF showing the 95% confidence intervals; LoF not shown as confidence intervals suggest age at onset not correlated with GMFCS risk. Calculated risk values (*right*) of GMFCS I and II (light blue), GMFCS III (blue), and GMFCS IV and V (dark blue) at different ages at onset at GoF variants.

Consistent with the strong association between GMFCS scores and the age at seizure onset, we designed a model by fitting the GMFCS score to the log_10_ of the age at seizure onset with an ordinal regression. The log transformation was chosen because the data were better approximated by a lognormal distribution. We describe this model as a simple risk‐prediction model due to the inherent assumptions in the model and the limited, but high‐quality, dataset to which we are fitting the model. The model fitted well to the data for GoF variants (*χ* ^2^ = 18.061, *p* = 2.1 × 10^−5^, Pearson goodness of fit *χ* ^2^ = 39.6, *p* = 0.762, Cox and Snell *R* ^2^ = 0.303). However, the model returns a poor fit to the age at onset of LoF variants, consistent with the lack of a robust association between GMFCS scores and the age at seizure onset (*χ* ^2^ = 2.685, *p* = 0.101, Pearson goodness of fit *χ* ^2^ = 85.8, *p* = 0.31, Cox and Snell *R* ^2^ = 0.045). Nevertheless, the risk of an individual with a LoF variant being non‐ambulatory (GMFCS IV and V) was less than for a patient with a GoF variant with any age at seizure onset (Figure 5A). The model predicts a very high likelihood (>90%) of an individual being non‐ambulatory when seizure onset is in the first month of life. This risk reduces with later age at seizure onset to ~35% by 20 months of age.

The risk of a milder level of motor dysfunction (GMFCS III) rises to about 31% and the risk of the mildest level of motor dysfunction (GMFCS I and II) rises to 35%. After 20 months of age, the risk of GMFCS IV and V and of III continues to decline (Figure 5).

## 4 DISCUSSION

In this study, we delineated the clinical trajectories of 117 individuals with *GABRB2‐* and *GABRB3*‐related disorders by comparing differences in epilepsy, cognitive, and motor function in individuals with LoF and GoF variants. Furthermore, we investigated the relationship between the age at seizure onset and the severity of gross motor dysfunction, ultimately developing a risk‐prediction model with utility for clinical diagnosis and prognosis of motor development and future treatment trials.

Our findings revealed distinctly different clinical trajectories for individuals with LoF or GoF *GABRB2* and *GABRB3* variants. In individuals with GoF variants, we identified more severe gross motor and cognitive impairment, together with earlier (often neonatal) seizure onset. By employing the age at seizure onset as a clinical biomarker, we developed a predictive model that forecasts gross motor function outcomes in individuals with GoF variants in *GABRB2* or *GABRB3*.

The age at seizure onset is a useful clinical biomarker that has been used previously as a proxy for the severity of the disorder.^8^ We reasoned that if the age at onset was a proxy for severity, it could also be used to predict the development of other morbidities. Therefore, we developed a simple risk model for gross motor deficits based on two variables: (1) whether the variant caused a loss or gain of function; and (2) the age at seizure onset.

We recently reported that individuals with *GABRB2* GoF variants more frequently had ongoing seizures, more severe epilepsy syndromes, more severe cognitive deficits, hyperkinetic movement disorders, and profound motor impairment (non‐ambulant) compared with those with LoF variants.^8^ However, in our previous study, the severity of gross motor function deficit was evaluated using a relatively coarse assessment (either reported by families or assessed ad hoc by treating clinicians), thereby limiting the ability to draw definitive conclusions about the relationship between gross motor outcomes and either the clinical phenotype or the functional consequence of the variant. By scoring gross motor dysfunction with the standardized GMFCS, we showed a high incidence of gross motor dysfunction in our GoF cohort.

Early diagnosis of gross motor dysfunction is important to prepare families and caregivers with realistic expectations regarding motor development and to ensure timely implementation of appropriate interventions. Because the age at seizure onset is a useful proxy for the overall severity of the disorder, we reasoned that it might provide a fast and reliable prediction of gross motor function compared with the challenges of deciphering functional properties of pathogenic variants. Indeed, gross motor outcomes strongly correlated with the age at seizure onset, and the simple risk model we developed shows that individuals with GoF variants who have seizure onset in the first month of life have an ~90% likelihood of never walking independently (GFMCS IV and V). This risk decreases with later seizure onset, with 30% of individuals with seizure onset by 20 months of age having GMFCS IV or V.

There are some limitations to our model. The first is that gross motor function outcomes are more severe in individuals with *GABRB3* than with *GABRB2* GoF variants. It is important to note that the age at seizure onset is also younger in the *GABRB3* cohort, and seizure onset in the first month of life occurs only in those with GoF variants. The second caveat is that the model fits an ordinal regression to the data that assumes proportional odds at the different severities of GMFCS. In both of these cases, the model will be less reliable for patients with later‐onset seizures with *GABRB3* variants, and in differentiating the risk between GMFCS I and II and GMFCS III, and hence why we frame it as a simple risk‐prediction model. However, neither of these caveats affect the main conclusion that the risk of being non‐ambulant, or scoring GMCFS IV–V, is very high when an individual with a de novo *GABRB2* or *GABRB3* variant presents with seizures in the first month of life. In practice, the functional motor outcome of these individuals, comprising ~10% of the overall cohort, can be confidently predicted without waiting for time‐consuming (and relatively inaccessible) functional analysis of the variant to be performed. Due to the cross‐sectional design of our study, we could not assess whether GMFCS levels remained stable in individuals over time.

We found that intellectual disability was diagnosed in the majority of individuals with a GoF variant; 82% had severe intellectual impairment. In contrast, severe intellectual disability was present in 11% of individuals with LoF variants. This is consistent with previously published *GABRB2* and *GABRB3* studies, where cognitive deficits were more frequent and severe in GoF cohorts.^5^, ^8^ Unlike gross motor dysfunction, the age at seizure onset did not correlate with the severity of cognitive deficits for individuals with LoF or GoF variants.

### 4.1 Strengths and limitations

This study includes a large number of participants, made possible by the support of families and treating physicians who enabled us to conduct in‐depth phenotypic analyses and assess outcomes. Because variant functional testing is available only in research settings, we provide a model that can predict the gross motor outcome of the variant in individuals with seizures. An important limitation of our study is selection bias, as patients with more severe epilepsies or neurodevelopmental delay are more likely to undergo molecular genetic testing. This could skew our data toward individuals with more severe clinical outcomes.

## 5 CONCLUSIONS

Our study advances understanding of the clinical trajectories of patients with *GABRB2‐* and *GABRB3*‐related disorders, providing valuable insights for families and physicians, as well as informing the design of future clinical trials. We found that gross motor dysfunction and cognitive deficits are more severe in individuals with GoF *GABRB2* or *GABRB3* variants. Using a simple risk model, clinicians can have high confidence in predicting the severity of gross motor dysfunction for individuals with *GABRB2* or *GABRB3* variants and seizure onset in the newborn or infantile period.

## AUTHOR CONTRIBUTIONS

S.O.: Methodology (Leading); conceptualization (Leading); data curation (Leading); writing‐original draft (Leading). L.A., C.B., R.S.D., A.F.H.H., S.A., A.B., S.Z., G.K., G.L., N.C., Z.G.S., M.T.P., M.A.P.T., E.S., A.S.M., S.B., M.A.O., S.T., H.A.D., P.B., A.R., N.Z., M.T., N.S., A.D., P.S., A.O., M.M.M., S.N., M.J.L., I.B.H., D.G., R.S., K.T., I.K., J.R.L., K.P., D.L., I.T., U.V., K.P.J.B., A.M.G., R.B., M.W., S.W., E.C., M.R.C., J.J., K.S., S.B., R.G., G.P., I.L.M., W.E.N., N.E.K., A.S., D.L.C., G.M., B.C., A.L., A.M., T.S., B.V.H., S.F.B., I.E.S., and E.G.: Writing – review & editing (equal). S.E.K., A.S.H.K., I.T.K., and P.K.A.: Functional testing, writing – review & editing (Equal). R.S.M. and N.L.A.: Methodology (Leading); conceptualization (Leading); data curation (Leading); writing – original draft (Leading).

## FUNDING INFORMATION

This study was supported by the Lundbeck Foundation (R383‐2022‐276: R.S.M., P.K.A.), the Australian Health and Medical Research Council (Ideas Grant APP2019780: P.K.A., R.S.M.; Investigator grants 1196637 S.F.B and 1172897 I.E.S, Synergy grant 2010562 S.F.B and I.E.S.)

## CONFLICT OF INTEREST STATEMENT

None of the authors have any conflicts of interest related to the manuscript being submitted for consideration in *Epilepsia*.

## ETHICAL PUBLICATION STATEMENT

We confirm that we have read the Journal's position on issues involved in ethical publication and affirm that this report is consistent with those guidelines.

## Supporting information


**TABLE S1.** Functional classification and clinical details for individuals carrying a *GABRB2* or *GABRB3* variant.

**TABLE S2.** American College of Medical Genetics (ACMG) scoring for variant classification of individuals carrying a *GABRB2* or *GABRB3* variant.

## Data Availability

Data are available upon reasonable request to the corresponding author.

## References

[1] Scheffer IE , Zuberi S , Mefford HC , Guerrini R , McTague A . Developmental and epileptic encephalopathies. Nat Rev Dis Primers. 2024;10(1):61. 39237642 10.1038/s41572-024-00546-6

[2] Oliver KL , Scheffer IE , Bennett MF , Grinton BE , Bahlo M , Berkovic SF . Genes4Epilepsy: an epilepsy gene resource. Epilepsia. 2023;64(5):1368–1375. 36808730 10.1111/epi.17547 PMC10952165

[3] Maljevic S , Møller RS , Reid CA , Pérez‐Palma E , Lal D , May P , et al. Spectrum of GABAA receptor variants in epilepsy. Curr Opin Neurol. 2019;32(2):183–190. 30664068 10.1097/WCO.0000000000000657

[4] Absalom NL , Lin SXN , Liao VWY , Chua HC , Møller RS , Chebib M , et al. GABAA receptors in epilepsy: elucidating phenotypic divergence through functional analysis of genetic variants. J Neurochem. 2024;168(12):3831–3852. 37621067 10.1111/jnc.15932 PMC11591409

[5] Absalom NL , Liao VWY , Johannesen KMH , Gardella E , Jacobs J , Lesca G , et al. Gain‐of‐function and loss‐of‐function GABRB3 variants lead to distinct clinical phenotypes in patients with developmental and epileptic encephalopathies. Nat Commun. 2022;13(1):1822. 35383156 10.1038/s41467-022-29280-x PMC8983652

[6] Johannesen KM , Iqbal S , Guazzi M , Mohammadi NA , Pérez‐Palma E , Schaefer E , et al. Structural mapping of GABRB3 variants reveals genotype–phenotype correlations. Genet Med. 2022;24(3):681–693. 34906499 10.1016/j.gim.2021.11.004

[7] Møller RS , Wuttke TV , Helbig I , Marini C , Johannesen KM , Brilstra EH , et al. Mutations in GABRB3: from febrile seizures to epileptic encephalopathies. Neurology. 2017;88(5):483–492. 28053010 10.1212/WNL.0000000000003565 PMC5278942

[8] Mohammadi NA , Ahring PK , Yu Liao VW , Chua HC , Ortiz De La Rosa S , Johannesen KM , et al. Distinct neurodevelopmental and epileptic phenotypes associated with gain‐ and loss‐of‐function GABRB2 variants. EBioMedicine. 2024;106:105236. 38996765 10.1016/j.ebiom.2024.105236 PMC11296288

[9] Absalom NL , Liao VWY , Kothur K , Indurthi DC , Bennetts B , Troedson C , et al. Gain‐of‐function *GABRB3* variants identified in vigabatrin‐hypersensitive epileptic encephalopathies. Brain Commun. 2020;2(2):fcaa162. 33585817 10.1093/braincomms/fcaa162 PMC7869430

[10] Musto E , Liao VW , Johannesen KM , Fenger CD , Lederer D , Kothur K , et al. GABRA1‐related disorders: from genetic to functional pathways. Ann Neurol. 2024;95(1):27–41. 10.1002/ana.26774 37606373

[11] Achkar CM , Harrer M , Smith L , Kelly M , Iqbal S , Maljevic S , et al. Characterization of the *GABRB2*‐associated neurodevelopmental disorders. Ann Neurol. 2021;89(3):573–586. 33325057 10.1002/ana.25985 PMC9161810

[12] Hamdan FF , Myers CT , Cossette P , Lemay P , Spiegelman D , Laporte AD , et al. High rate of recurrent de novo mutations in developmental and epileptic encephalopathies. Am J Hum Genet. 2017;101(5):664–685. 29100083 10.1016/j.ajhg.2017.09.008 PMC5673604

[13] Maillard P , Baer S , Schaefer É , Desnous B , Villeneuve N , Lépine A , et al. Molecular and clinical descriptions of patients with GABA_A_ receptor gene variants (*GABRA1, GABRB2, GABRB3, GABRG2*): a cohort study, review of literature, and genotype–phenotype correlation. Epilepsia. 2022;63(10):2519–2533. 35718920 10.1111/epi.17336 PMC9804453

[14] Burgess R , Wang S , McTague A , Boysen KE , Yang X , Zeng Q , et al. The genetic landscape of epilepsy of infancy with migrating focal seizures. Ann Neurol. 2019;86(6):821–831. 31618474 10.1002/ana.25619 PMC7423163

[15] Stödberg T , Tomson T , Barbaro M , Stranneheim H , Anderlid B , Carlsson S , et al. Epilepsy syndromes, etiologies, and the use of next‐generation sequencing in epilepsy presenting in the first 2 years of life: a population‐based study. Epilepsia. 2020;61(11):2486–2499. 32964447 10.1111/epi.16701 PMC7756847

[16] Pavone P , Pappalardo XG , Marino SD , Sciuto L , Corsello G , Ruggieri M , et al. A novel *GABRB3* variant in Dravet syndrome: case report and literature review. Mol Genet Genomic Med. 2020;8(11):e1461. 32945607 10.1002/mgg3.1461 PMC7667356

[17] Yang Y , Xiangwei W , Zhang X , Xiao J , Chen J , Yang X , et al. Phenotypic spectrum of patients with *GABRB2* variants: from mild febrile seizures to severe epileptic encephalopathy. Dev Med Child Neurol. 2020;62(10):1213–1220. 32686847 10.1111/dmcn.14614

[18] Štěrbová K , Vlčková M , Klement P , Neupauerová J , Staněk D , Zůnová H , et al. Neonatal onset of epilepsy of infancy with migrating focal seizures associated with a novel GABRB3 variant in monozygotic twins. Neuropediatrics. 2018;49(03):204–208. 29444535 10.1055/s-0038-1626708

[19] Hirsch E , French J , Scheffer IE , Bogacz A , Alsaadi T , Sperling MR , et al. ILAE definition of the idiopathic generalized epilepsy syndromes: position statement by the ILAE Task Force on Nosology and Definitions. Epilepsia. 2022;63(6):1475–1499. 35503716 10.1111/epi.17236

[20] Riney K , Bogacz A , Somerville E , Hirsch E , Nabbout R , Scheffer IE , et al. International League Against Epilepsy classification and definition of epilepsy syndromes with onset at a variable age: position statement by the ILAE Task Force on Nosology and Definitions. Epilepsia. 2022;63(6):1443–1474. 35503725 10.1111/epi.17240

[21] Specchio N , Wirrell EC , Scheffer IE , Nabbout R , Riney K , Samia P , et al. International League Against Epilepsy classification and definition of epilepsy syndromes with onset in childhood: position paper by the ILAE Task Force on Nosology and Definitions. Epilepsia. 2022;63(6):1398–1442. 35503717 10.1111/epi.17241

[22] Zuberi SM , Wirrell E , Yozawitz E , Wilmshurst JM , Specchio N , Riney K , et al. ILAE classification and definition of epilepsy syndromes with onset in neonates and infants: position statement by the ILAE Task Force on Nosology and Definitions. Epilepsia. 2022;63(6):1349–1397. 35503712 10.1111/epi.17239

[23] Beniczky S , Trinka E , Wirrell E , Specchio N , Cendes F , Cross JH . Updating the ILAE seizure classification. Epilepsia. 2025;66(6):1824–1826. 10.1111/epi.18399 40264360

[24] Palisano R , Rosenbaum P , Walter S , Russell D , Wood E , Galuppi B . Development and reliability of a system to classify gross motor function in children with cerebral palsy. Dev Med Child Neurol. 1997;39(4):214–223. 9183258 10.1111/j.1469-8749.1997.tb07414.x

[25] Rossi A , Lin SXN , Absalom NL , la Ortiz‐De Rosa S , Liao VWY , Mohammadi NA , et al. Phenotypic spectrum in individuals with pathogenic GABRG2 loss‐ and gain‐of‐function variants. Neurology. 2025;105(2):e213644. 40570274 10.1212/WNL.0000000000213644 PMC12202131

[26] Richards S , Aziz N , Bale S , Bick D , Das S , Gastier‐Foster J , et al. Standards and guidelines for the interpretation of sequence variants: a joint consensus recommendation of the American College of Medical Genetics and Genomics and the Association for Molecular Pathology. Genet Med. 2015;17(5):405–424. 25741868 10.1038/gim.2015.30 PMC4544753

[27] Diagnostic and statistical manual of mental disorders Text Revision: DSM‐5‐TR. 5th ed. Washington, DC: American Psychiatric Association; 2022.

